# Effect of pH on compressive strength of some modification of mineral trioxide aggregate

**DOI:** 10.4317/medoral.18922

**Published:** 2013-05-31

**Authors:** Mohammad A. Saghiri, Franklin Garcia-Godoy, Armen Asatourian, Mehrdad Lotfi, Sepideh Banava, Kaveh Khezri-Boukani

**Affiliations:** 1 BSc, MSc, PhD Assistant Professor, Department of Dental Material, Dental School, Member of Craniofacial Research center, Azad University (Tehran Branch) and Kamal Asgar Research Center (KARC) Tehran, Iran; 2 DDS, MS, PhD Professor, Bioscience Research Center, College of Dentistry, University of Tennessee Health Science Center, Memphis, TN, USA; 3 DDS visiting scientist, Kamal Asgar Research Center (KARC), Tehran, Iran; 4 DMD, MSc Professor, Research Center for Pharmaceutical Nanotechnology and Department of Endodontics, Dental Faculty, Tabriz University (Medical Sciences), Tabriz, Iran; 5 DMD, MSc Head and Assistant Professor, Department of Dental Material, Islamic Azad University (Tehran Branch), Tehran, Iran; 6 BSc, MSc Islamic Azad University, Tehran, Iran

## Abstract

Objectives: Recently, it was shown that NanoMTA improved the setting time and promoted a better hydration process which prevents washout and the dislodgment of this novel biomaterial in comparison with WTMA. This study analyzed the compressive strength of ProRoot WMTA (Dentsply), a NanoWMTA (Kamal Asgar Research Center), and Bioaggregate (Innovative Bioceramix) after its exposure to a range of environmental pH conditions during hydration. Study Design: After mixing the cements under aseptic condition and based on the manufacturers` recommendations, the cements were condensed with moderate force using plugger into 9 × 6 mm split molds. Each type of cement was then randomly divided into three groups (n=10). Specimens were exposed to environments with pH values of 4.4, 7.4, or 10.4 for 3 days. Cement pellets were compressed by using an Instron testing machine. Values were recorded and compared. Data were analyzed by using one-way analysis of variance and a post hoc Tukey’s test. Results: After 3 days, the samples were solid when probed with an explorer before removing them from the molds. The greatest mean compressive strength 133.19±11.14 MPa was observed after exposure to a pH value of 10.4 for NanoWMTA. The values decreased to 111.41±8.26 MPa after exposure to a pH value of 4.4. Increasing of pH had a significant effect on the compressive strength of the groups (p<0.001). The mean compressive strength for the NanoWMTA was statistically higher than for ProRoot WMTA and Bioaggregate (p<0.001). Moreover, increasing of pH values had a significant effect on compressive strength of the experimental groups (p<0.001). Conclusion: The compressive strength of NanoWMTA was significantly higher than WMTA and Bioaggregate; the more acidic the environmental pH, the lower was the compressive strength.

** Key words:**Compressive strength, mineral trioxide aggregate, Nano.

## Introduction

Mineral Trioxide Aggregate (MTA) showed better psychochemical properties including bioactivity and setting in the presence of moister in comparison with other similar materials (1). However, MTA has some disadvantages, such as prolonged setting time (1) , poor handling attributes (2) , absence of a known solvent for this material, and the dif?culty of its removal after setting (3). Several studies made modifications to improve the properties of MTA, but some of them could not show effectiveness as it was expected (4).

Bioaggregate (BA; Innovative BioCeramix, Vancouver, Canada), a calcium silicate–based material, is a modi?ed type of MTA (5). Most of the constituents are the same as that in white MTA (WMTA) except that BA is aluminum free (6). MTA and BA have same antibacterial behaviour (7), similar ability to prevent leakage (8) and same cell toxicity (6); however the push-out strength in furcal perforations repaired with WMTA was superior than those for BA in acidic condition; both cements are adversely affected by acidic pH (9) but there is no published study reporting the compressive strength of BA.

pH changes produce different behaviors of MTA in various conditions; when MTA comes into contact with existing in?amed tissues it may be exposed to a low pH (as low as 5) (10) , which can affect the sealing and physicochemical properties of MTA producing reduced bond strength to dentin (11). In contrast, higher pH (pH 8.4) enhanced the MTA-dentin bond strength (12) and decreased leakage (13). The low pH of the surrounding tissues also affected the hydration reaction of MTA (14). Acidic environment reduced the surface hardness of MTA (10,15,16) in combination with reduced compressive (17) and diametral strength (16). However, the hardness of MTA could be improved in a pH of 8.4 to 9.4 (18).

Moreover, compressive strength is an important factor when a material is placed in a cavity, which bears occlusal pressures in some applications like direct pulp capping (19), vital pulp therapies (20), and the placement of coronal barrier (21). Torabinjead et al. reported that the compressive strength of MTA increased during 3 weeks after placement in moist environment containing distilled water (22); meanwhile, other investigators have indicated that the amount of microleakage can be reduced when MTA is in contact with synthetic tissue fluid (13).

To accelerate the setting time and to speed up the hydration procedure of WMTA (23) and Portland cement, the size of particles have been made smaller (24). A new type of MTA in nano-size scale (NWMTA) has been recently patented in the USA (US Pat-ent application No 13/211.880) with new characteristics such as fast setting time (less than 5 minutes). An investigation (25) showed its efficacy in physical and chemical changes which attributes to its increased surface area, resistance to acidic environment and less porosity.

The aim of present study was to compare the compressive strength of WMTA, BA and NWMTA in acidic and alkaline pH conditions. The null hypothesis was tested that the compressive strength value of tested cements would not be affected significantly when they were exposed to either acidic or alkaline situation.

## Material and Methods

**Figure 1:**
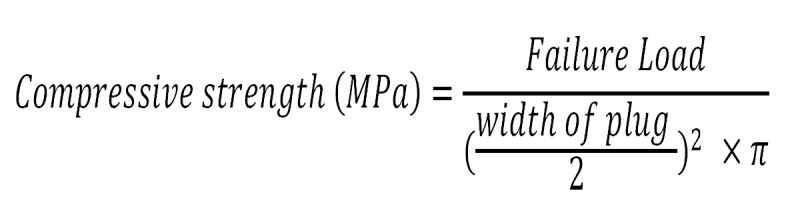
Formula for calculating the resistance.

The compressive strengths of WMTA (Tooth-colored formula, ProRoot MTA, Dentsply, Tulsa, OK) and Bioaggregate (BA; Innovative BioCeramix, Vancouver, Canada) and Nano White Mineral Trioxide Aggregate (NWMTA; Kamal Asgar Research Center, US Pat # 13/211880 ) were determined according to the method recommended by the ISO 6876 (26). In brief, each material was mixed according the manufacturers’ instructions and placed in one-end closed split stainless steel molds with 9 mm high and 6 mm wide. Thirty samples of each material were prepared and randomly divided into 3 groups of 10 specimens (n=10) and exposed to different pH values. Synthetic tissue fluid (STF), prepared as 1.7 g of KH2PO4, 11.8 g of Na2HPO4, 80.0 g of NaCl, and 2.0 g of KCl was prepared in pH 7.4, in pH 4.4 with butyric acid and pH 10.4 with potassium hydroxide. Then, 10 samples of each material were wrapped in pieces of gauze soaked in STF buffered in butyric acid and potassium hydroxide at pH 4.4 (group A for each material), 10 specimens at pH 7.4 (group B for each material ) and finally 10 for pH 10.4 (group C for each material), respectively. The pieces of gauze were refreshed every 24 hours (daily) to stabilize pH values (12,18,25). The whole assembly was placed in an incubator with a constant temperature of 37°C and 95% humidity for 3 days (12,18,25). Specimens were removed from the molds and placed lengthwise between the platens of an Instron 85215 (Instron Corp, Canton, MA) testing machine. All of the samples were solid when probed with an explorer before removing them from the molds. The samples were compressed at a rate of 1 millimeter/minutes, and the load at fracture was recorded in megapascals (MPa). Two specimens of BA in pH of 4.4 subgroups failed after being placed lengthwise between the platens of an Instron machine, underwent complete breakdown due to low mechanical strength therefore they were excluded from study. To calculate the compressive strength the formula below was used, using the values for height, diameter, weight and failure load. (Fig. 1).

One-way analysis of variance with a post hoc Turkey test was used to determine statistical differences among compressive strengths of the tested materials.

## Results

**Figure 2:**
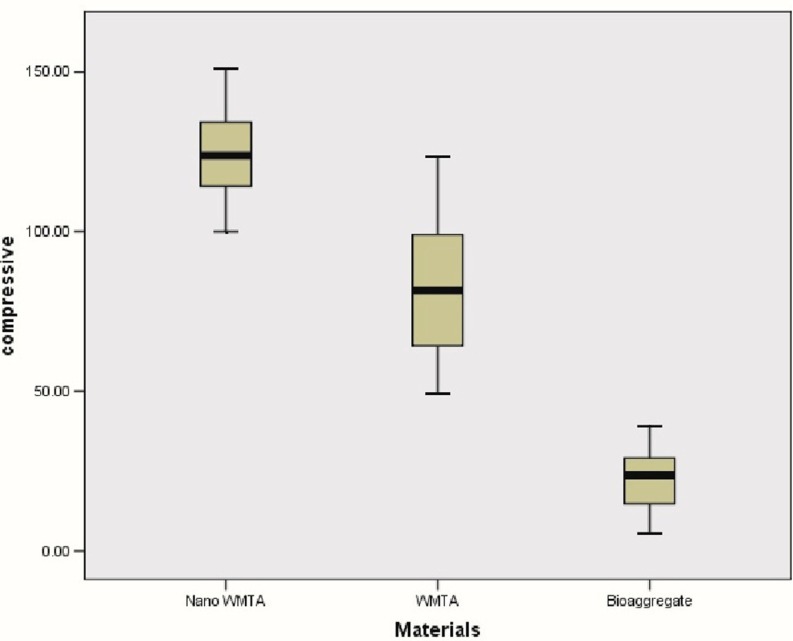
Box plots of compressive strength (MPa) values for each type of cement as overall showing the axial amount of force for the compressive strength reached to crush the cement plug.

The means value of compressive strength for experimental groups as following:

- WMTA: 63.78 (pH=4.4), 86.23 (pH=7.4), and 103.63 (pH=10.4).

- Bioaggregate: 10.88 (pH=4.4), 25.36 (pH=7.4), and 29.07 (pH=10.4).

- NanoWMTA: 111.41 (pH=4.4), 126.81 (pH=7.4), and 133.19 (pH=10.4). (Fig. 2)

**Figure 3:**
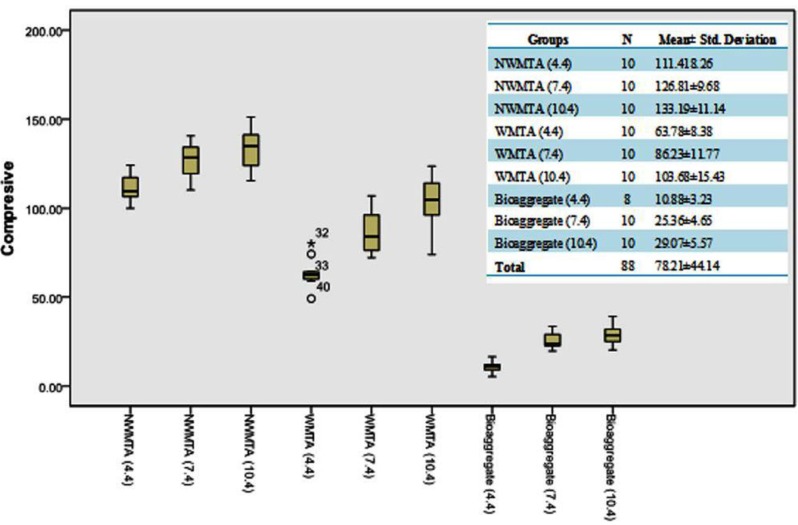
Box plots of the compressive strength of each sub group of WMTA, Bioaggregate, and NanoWMTA in contact with different pH values.

The greatest mean compressive strength 133.19±11.14 MPa was observed after exposure to a pH value of 10.4 for NanoWMTA; however, the values decreased to 111.41±8.26 MPa after exposure to a pH of 4.4. Increasing the pH had significant effects on the compressive strength of the all experimental groups (p<0.001). The mean compressive strength for the NanoWMTA was significantly more than WMTA and Bioaggregate (p<0.001) ( Table 1). Meanwhile, the statistical analyses revealed a significant interaction between the increase of the pH value and the compressive strength for all tested materials (p<0.001) (Fig. 3).

**Table 1:**
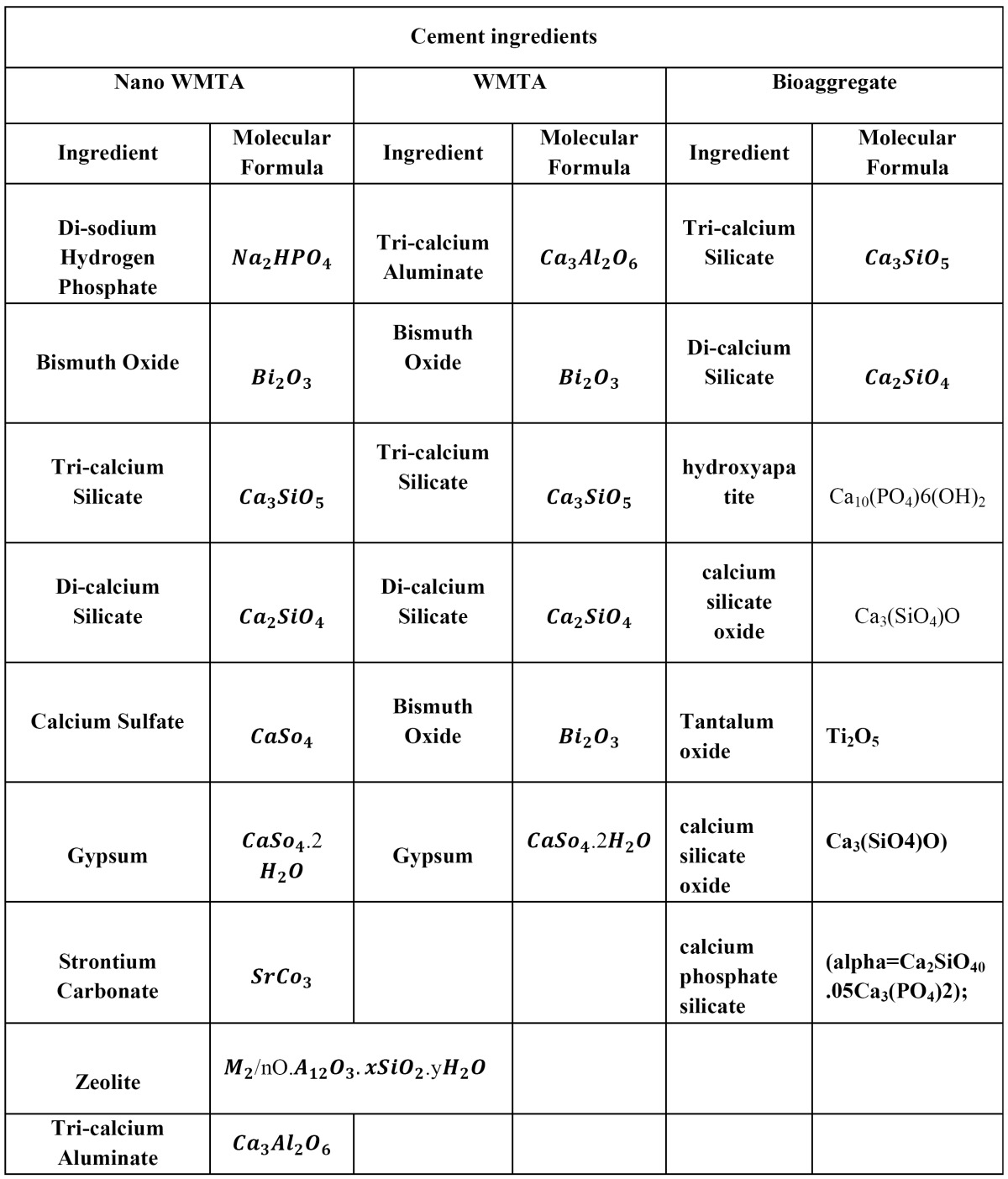
The ingredients of WMTA, Nano WMTA and Bioaggregate (4,25,36).

## Discussion

Nano-structured materials are de?ned as having a grain size not exceeding 100 nm, typically a range between 5 to 50 nm. Nano-structure addresses one of the key problems of endodontic cements such as setting time (27). Although the detailed mechanism of reaction of nano size powder needs to be determined for each speci?c cement system, experiments indicated that in practically all nano powders (whether oil- or water-based reaction), the kinetics of both absorption and desorption can be improved by the order of magnitude simply by reducing the grain size of the powder (28).

In the present study, we analyzed the compressive strength of two in comparison with new NanoWMTA which has been recently patented (27). By considering the wide ranges of application of MTA (21) this factor plays an important role in the success of the treatment in some indications of this cement such as furcation perforations in which occlusal forces can be directly exposed to the applied cement (9). On the other hand, the long setting time of MTA can lead to additional costs when adding different additives such as CaCl2 (29) or Na2HPO4 (30) to prevent further washing-out and dislodgment (21). We examined the compressive strength of these three materials in three different conditions; acidic, neutral and alkaline environments. STF was used as a solution to mimic the physiological situation. In presence of infection and inflammation, the pH of tissue can be reduced (31). Furthermore, the pH value of most local anesthetics in cartridge form is purposefully low (pH3½-4), because the charged, acid form of the molecule is more stable (as is the vasoconstrictor) at a low pH, and thus provides a longer shelf life (32). Therefore, butyric acid was used in the present study to simulate the effect of by products of anaerobic bacterial metabolism (33) and stimulate clinical scenario. On the other hand, when MTA is used as an apical plug in open apex cases after the removal of calcium hydroxide, they may be placed in an alkaline pH environment and may alter the properties of MTA. Furthermore, all samples were condensed with a hand condenser and hand pressure due to the effectiveness of this method to prevent the formation of more voids (34).

Our results showed that acidic conditions had adverse effects in the compressive strength of these cements. Many studies reported similar results and the interference of acidic environment in the setting procedure (35), reduced strength (25), hardness, and the porosity of MTA (10,13). BA had the lowest values in all pH conditions in comparison with WMTA and NWMTA. BA has very similar composition than MTA (36), but it is aluminum free and had different opaquers. Different radiopaquers can change the physical and chemical behavior of MTA (37) and decrease the compressive strength (38). Hashem et al. have investigated the dislodgement resistance of BA and MTA applied in furcation perforations (9). These authors have indicated that BA had less resistance in acidic situation in comparison with MTA cement; however, MTA was more influenced by acidic pH values than BA (9), which can suggest that BA cement is less applicable material especially in cases where occlusal forces are expected. The results showed an upward trend of the compressive strength values in each material in higher pH conditions and the greatest compressive strength values were observed in NWMTA at different pH values, 4.4, 7.4, 10.4, among others with 111.166, 126.82 and 133.19 MPa, respectively. BA had the lowest compressive strength particularly in pH 4.4 around 10.88 MPa. In acidic condition, NanoWMTA exhibited the highest compressive value in comparison with other tested materials. The present study revealed that the greatest mean of compressive strength values were observed after exposure to a pH of 10.4. This finding is partly in agreement with the results of previous studies on the effect of alkaline pH on microhardness (18) and push bond strength of WMTA (12) which showed the higher surface hardness as well as less porosity and unhydrated structure in alkaline pH values. MTA might be exposed to a high-pH environment during treatment. Shokouhinejad et al. (11) showed an increase in the mean of push-out bond strength of WMTA as the pH was raised from 4.4 to 7.4, which is consistent with the present results as the compressive strength of WMTA increased following exposure to pH 7.4 compared with pH 4.4. Tronstad et al. (39) showed that the pH of the most inner part of the circumpulpal dentin could chang the pH of its nearby environment from 11.1 to 12.2 after placing calcium hydroxide in the root canals.

The current study supports the previous study (25) stating that NWMTA had less porosity, better hydration, and good interlocking crystals than WMTA even in an acidic pH. The present study also elucidated that NWMTA also kept its high strength in acidic environment compared to WMTA and BA. Therefore, pH alterations can jeopardize the structure of WMTA that may increase/decrease compressive strength. NanoWMTA showed better compressive strength value in acidic environments, however further studies regarding the cell interaction and biological behavior of this nano structure new cement are needed to address biological and bioactive responses.

## Conclusion

According to present in-vitro study, following conclusions have been drawn.

• Acidic environment can drastically affect the compressive strength values of all tested materials. However, the increasing of pH in alkaline environment can enhance this property significantly in mentioned cements.

• In low pH conditions unfavorable structural changes can occur in the microsturcutre of cement materials which caused higher porosity in applied substances and eventually affect their compressive strength negatively.

• NanoWMTA due to its higher compressive strength observed in all pH conditions can be suggested as a remarkable root end filling material, especially in situations in which the applied material might be exposed to acidic environments.
